# Diesel exhaust particles induce CYP1A1 and pro-inflammatory responses via differential pathways in human bronchial epithelial cells

**DOI:** 10.1186/1743-8977-7-41

**Published:** 2010-12-16

**Authors:** Annike I Totlandsdal, Flemming R Cassee, Per Schwarze, Magne Refsnes, Marit Låg

**Affiliations:** 1 Department of Air Pollution and Noise, Division of Environmental Medicine, Norwegian Institute of Public Health, Oslo, Norway; 2 National Institute for Public Health and the Environment, Bilthoven, the Netherlands

## Abstract

**Background:**

Exposure to diesel engine exhaust particles (DEPs) has been associated with several adverse health outcomes in which inflammation seems to play a key role. DEPs contain a range of different inorganic and organic compounds, including polycyclic aromatic hydrocarbons (PAHs). During the metabolic activation of PAHs, CYP1A1 enzymes are known to play a critical role. In the present study we investigated the potential of a characterised sample of DEPs to induce cytotoxicity, to influence the expression of CYP1A1 and inflammation-related genes, and to activate intracellular signalling pathways, in human bronchial epithelial cells. We specifically investigated to what extent DEP-induced expression of interleukin (IL)-6, IL-8 and cyclooxygenase (COX)-2 was regulated differentially from DEP-induced expression of CYP1A1.

**Results:**

The cytotoxicity of the DEPs was characterised by a marked time- and concentration-dependent increase in necrotic cells at 4 h and above 200 μg/ml (~ 30 μg/cm^2^). DEP-induced DNA-damage was only apparent at high concentrations (≥ 200 μg/ml). IL-6, IL-8 and COX-2 were the three most up-regulated genes by the DEPs in a screening of 20 selected inflammation-related genes. DEP-induced expression of CYP1A1 was detected at very low concentrations (0.025 μg/ml), compared to the expression of IL-6, IL-8 and COX-2 (50-100 μg/ml). A CYP1A1 inhibitor (α-naphthoflavone), nearly abolished the DEP-induced expression of IL-8 and COX-2. Of the investigated mitogen-activated protein kinases (MAPKs), the DEPs induced activation of p38. A p38 inhibitor (SB202190) strongly reduced DEP-induced expression of IL-6, IL-8 and COX-2, but only moderately affected the expression of CYP1A1. The DEPs also activated the nuclear factor-κB (NF-κB) pathway, and suppression by siRNA tended to reduce the DEP-induced expression of IL-8 and COX-2, but not CYP1A1.

**Conclusion:**

The present study indicates that DEPs induce both CYP1A1 and pro-inflammatory responses in vitro, but via differential intracellular pathways. DEP-induced pro-inflammatory responses seem to occur via activation of NF-κB and p38 and are facilitated by CYP1A1. However, the DEP-induced CYP1A1 response does not seem to involve NF-κB and p38 activation. Notably, the present study also indicates that expression of CYP1A1 may represent a particular sensitive biomarker of DEP-exposure.

## Background

Exposure to particulate matter (PM) in ambient air has been linked to adverse cardiopulmonary effects in epidemiological studies [1,2]. The biological mechanisms explaining these associations are currently not clarified, but inflammation is considered as a key event. Emissions from motor vehicles contribute significantly to urban particulate air pollution [3], and will despite regulations probably continue to do so, due to the general increase in intensity of and reliance on transport. In addition, there has been a tremendous increase in the use of diesel cars in Europe, which compared to petrol-fuelled cars have been known to emit more PM per kilometre. As a consequence, health effects of diesel engine exhaust particles (DEPs) have been studied intensively and will continue to be of interest to study, also in the evaluation of new emission control strategies.

DEPs represent a variable and complex mixture which may contain a range of different organic and inorganic compounds. Polycyclic aromatic hydrocarbons (PAHs) represent one such group of components and have been identified as potentially important contributors to the health effects associated with exposure to combustion particles, including DEPs [4]. CYP1A1-enzymes play a critical role in the metabolic activation of PAHs, and are highly inducible by PAHs via aryl hydrocarbon receptor (AhR)-mediated gene transcription [5]. The potency of DEPs to induce gene expression of CYP1A1 has previously been demonstrated by DEP-extract in human lung samples *ex vivo *[6] and by DEPs as well as DEP-extracts in human airway epithelial (16HBE) and human macrophage (U937) cell lines [7,8].

Cellular expression of genes may involve the activation of a range of intracellular transduction pathways. The present paper focuses on DEP-induced activation of mitogen-activated protein kinases (MAPKs) and nuclear factor-κB (NF-κB). Activation of these important signalling pathways has been detected in biopsies of lung tissue from humans exposed to diesel exhaust [9] and in *in vitro *cell models [7,10,11]. However, the involvement of these pathways in DEP-induced CYP1A1 expression, in relation to pro-inflammatory genes, remains to be determined. Several studies on the regulation of AhR indicate that toxic responses induced by AhR ligands, such as PAHs, occur through changes in cellular oxidative status that may alter the activities of transcription factors involved in the oxidative stress response [12]. Among such redox-sensitive transcription factors, it has been demonstrated that NF-κB and AP-1 cross-talk with AhR that modulates the expression of its regulated genes [13]. Thus, NF-κB, AP-1 and associated MAPK signaling pathways may play a crucial role in the regulation of AhR and its dependent genes.

Our group has recently demonstrated that benzo[a]pyrene (B[a]P) induced expression of CYP1A1, but not cytokine/chemokine responses in BEAS-2B cells [14]. In the present study the CYP1A1-response of these cells was studied in more detail upon exposure to DEPs, containing B[a]P in addition to several other PAHs. Our main hypothesis was that CYP1A1 expression might influence the DEP-induction of pro-inflammatory mediators. The CYP1A1 response was therefore studied in relation to the regulation of DEP-induced expression of selected inflammation-related genes. Furthermore, we examined to what extent differential intracellular pathways were involved in the DEP-induced expression of CYP1A1 and selected inflammation-related genes.

## Results

### DEP-induced cytotoxicity

**Figure 1:**
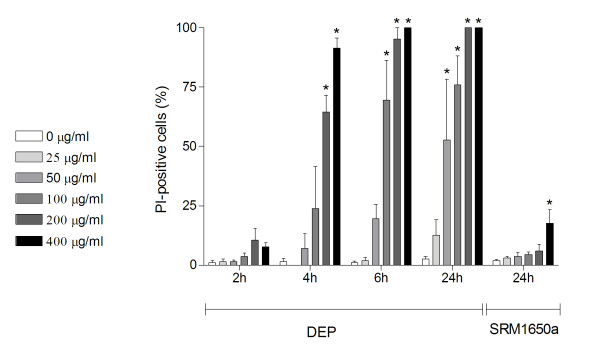
**Time- and concentration-dependent DEP-induced and concentration-dependent SRM1650a-induced cytotoxicity**. Human bronchial epithelial BEAS-2B cells were exposed to increasing concentrations of DEPs (0-400 μg/ml) for 2, 4, 6 or 24 h, before they were stained with Hoechst 33342 and PI and analysed by fluorescence microscopy. SRM1650a served as a reference sample (0-400 μg/ml) for cytotoxicity induced after 24 h of exposure. Bars represent means ± SEM of the percentage of PI-positive cells counted in separate experiments (n = 3). * p < 0.05; exposed vs. unexposed cells at each investigated timepoint.

The DEPs used in the present study were relatively cytotoxic, compared to the commercially available Standard Reference Diesel Material (SRM) 1650a. Microscopic analysis after propidium iodide (PI) and Hoechst staining of the particle-exposed cells revealed that the cytotoxicity primarily was characterised by a concentration-dependent increase in necrotic cells, especially at 6 and 24 h (Figure 1). At 24 h, the DEPs induced a cytotoxic response at 50 μg/ml (~50% PI-positive cells), with maximal toxicity at 200 μg/ml (100% PI-positive cells). In comparison, the toxicity of SRM 1650a was observed first at 400 μg/ml, with ~15% PI-positive cells. A slight increase in apoptotic cells was detected in cells exposed to DEPs for 24 h, but the percentage of apoptotic cells always remained below 5% (data not shown). The standard reference diesel material was not included in further experiments.

### Multiple gene expression analysis of DEP-exposed cells

**Figure 2:**
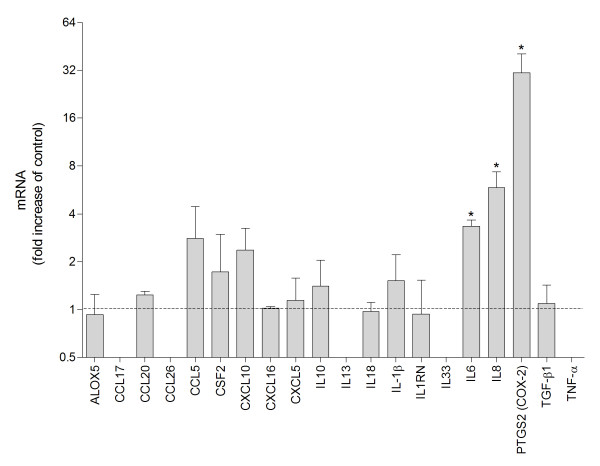
**DEP-induced mRNA expression of multiple inflammation-related genes**. Human bronchial epithelial BEAS-2B cells were exposed to 100 μg/ml DEPs for 4 h. Relative quantification of mRNA levels was performed by QRT-PCR. Bars represent means ± SEM of the relative change compared to control cells after normalization for beta-actin mRNA levels, detected in three independent experiments. * more than two-fold increase compared to control (dotted line) in all three experiments.

The DEPs were examined for their effects on the expression of 20 different inflammation-related genes by quantitative real-time (QRT)- PCR. The genes were mainly selected based on a previous study in which the effects of PAHs and other components commonly associated with particulate air pollution were tested [15]. After exposure to DEP (100 μg/ml) for 4 h, IL-6, IL-8 and COX-2 appeared to be most up-regulated with an approximately 3-fold, 6-fold and 30-fold average increase, respectively (Figure 2). Among the other studied genes only CCL5 and CXCL10 were on average up-regulated more than 2-fold. However, in contrast to IL-6, IL-8 and COX-2, expression of these genes was only increased more than two-fold in two out of three experiments.

### DEP-induced expression of selected genes (IL-6, IL-8, COX-2 and CYP1A1)

**Figure 3:**
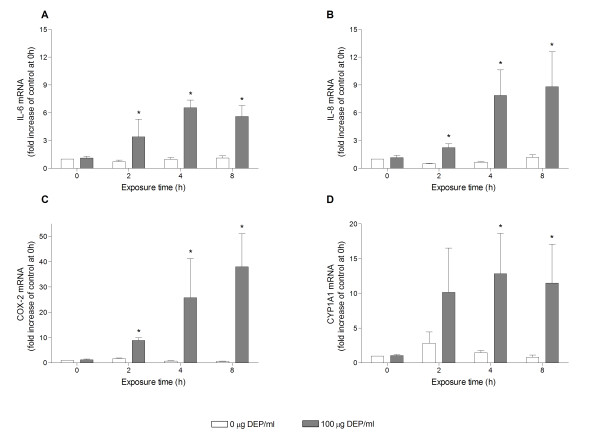
**Time-dependent DEP-induced expression of IL-6 (A), IL-8 (B), COX-2 (C) and CYP1A1 (D) mRNA**. Human bronchial epithelial BEAS-2B cells were exposed to 0 or 100 μg/ml DEPs for 0, 2, 4 or 8 h. Relative quantification of cytokine mRNA levels was performed by QRT-PCR. Bars represent means ± SEM of fold increase relative to unexposed cells at 0 h, detected in separate experiments (n≥3). * p < 0.05 exposed vs. unexposed cells.

Based on the results from the multiple gene expression analysis, the DEP-induced expression of IL-6, IL-8 and COX-2 was investigated in further detail, in addition to the expression of CYP1A1. Time course experiments indicated that the mRNA levels of IL-6, IL-8 and COX-2 are increasingly up-regulated by DEPs (100 μg/ml) with time, reaching their maxima at 4 h (IL-6 and IL-8) and at 8 h (COX-2) (Figure 3). In contrast, the CYP1A1 expression occurred at early time-points with a pronounced increase at 2 h, and a levelling off at 4 and 8 h. At 24 h, mRNA levels of all four genes were lower than the levels observed at 8 h (data not shown).

**Figure 4:**
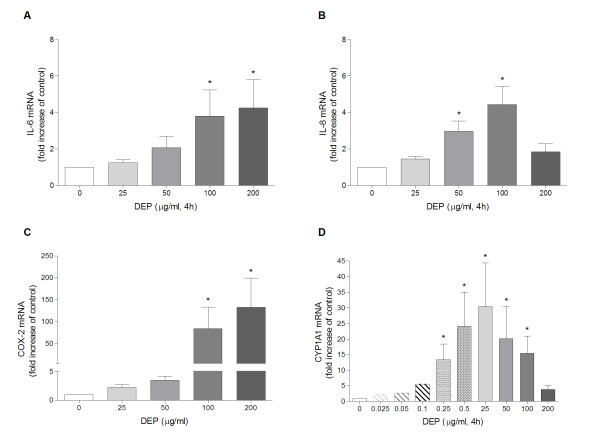
**Concentration-dependent DEP-induced expression of IL-6 (A), IL-8 (B), COX-2 (C) and CYP1A1 (D) mRNA**. Human bronchial epithelial BEAS-2B cells were exposed to increasing concentrations of DEPs (0-200 μg/ml) for 4 h. Relative quantification of cytokine mRNA levels was performed by QRT-PCR. Bars represent means ± SEM of fold increase relative to unexposed cells detected in separate experiments (n = 3). Experiments with very low concentrations (0.025, 0.05 and 0.1 μg/ml) were only repeated twice. * p < 0.05; exposed vs. unexposed cells.

The concentration-dependent increase in the mRNA levels of these genes was investigated after 4 h exposure to DEPs. The mRNA levels of IL-6, IL-8 and COX-2 appeared to increase at 25 μg/ml and to reach their maxima at 100-200 μg/ml DEPs (Figure 4). Notably, the average DEP-induced increase in COX-2 expression (~125-fold increase) was of a much higher magnitude than for IL-6 and IL-8 (~5-fold increase). Compared to the DEP-induced expression of IL-6, IL-8 and COX-2, the CYP1A1 expression increased at much lower DEP-concentrations. Expression of CYP1A1 was induced by DEPs already at a concentration of 0.025-0.05 μg/ml, and reached a maximum at 0.5-25 μg/ml (Figure 4). The decrease in CYP1A1 expression at higher concentrations (50 μg/ml) seemed to coincide with an increase in the expression of IL-6, IL-8 and COX-2.

### DEP-induced release of IL-6 and IL-8

**Figure 5:**
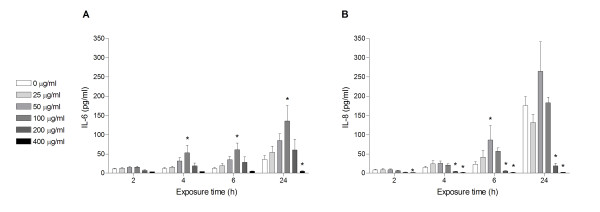
**Time- and concentration-dependent release of IL-6 (A) and IL-8 (B)**. Human bronchial epithelial BEAS-2B cells were exposed to increasing concentrations of DEPs (0-400 μg/ml) for 2, 4, 6 or 24 h. Cytokine concentrations were determined by ELISA. Histograms represent means ± SEM of separate experiments (n = 3). * p < 0.05; exposed vs. unexposed cells at each time point.

DEP-induced release of IL-6 and IL-8 was investigated by enzyme-linked immunoabsorbent assay (ELISA) analysis. A time-as well as concentration-dependent DEP-induced increase in the release of IL-6 and IL-8 was detected (Figure 5). Maximum levels of IL-6 and IL-8 were reached after 24 h exposure, at DEP concentrations of 100 and 50 μg/ml, respectively. Cytokine levels decreased with further increase in DEP-concentrations. Similar patterns were also apparent at earlier time points (4 h and 6 h). Although not statistical significant, DEP-induced increases in IL-6 and IL-8 release were detected in all repetitive experiments already after 4 hours in cells exposed to 50 μg/ml. In general, the relative increase of DEP-induced release was more pronounced for IL-6 than for IL-8.

### DEP-induced activation of intracellular signalling pathways

**Figure 6:**
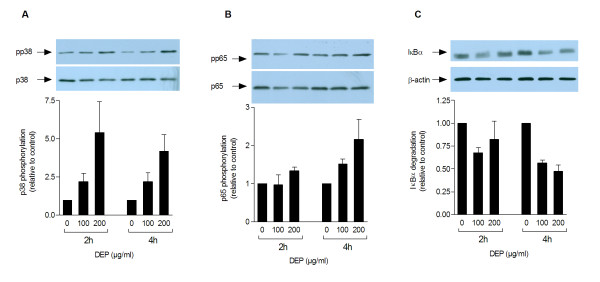
**DEP-induced phosphorylation of p38 (A), phosphorylation of p65 (B) and degradation of IκBα (C)**. Human bronchial epithelial BEAS-2B cells were exposed to increasing concentrations of DEPs (0, 100, 200 μg/ml) for 2 or 4 h before Western analysis. Each figure displays a representative blot and optical quantification of the protein bands from separate replicate experiments. Detected levels of phosphorylated p38 and p65 and the levels of IκBα are respectively normalised against total p38, p65, or β-actin, and are presented as fold increase (mean ± SEM, n≥3) compared to unexposed cells.

DEP-induced activation of intracellular signalling pathways was investigated by Western analysis. In cell cultures incubated with DEPs, phosphorylation of p38 increased with higher concentrations (0, 100 and 200 μg/ml) at 2 and 4 h (Figure 6A). No DEP-induced increase in the phosphorylation of ERK and JNK was detected (data not shown). DEP-induced activation of NF-κB was evaluated by examining p65 phosphorylation and IκBα degradation. DEP-induced phosphorylation of p65 and degradation of IκBα was most evident at 4 h (Figure 6B, C).

### Differential effects of inhibitors on DEP-induced expression of IL-6, IL-8, COX-2 and CYP1A1

**Figure 7:**
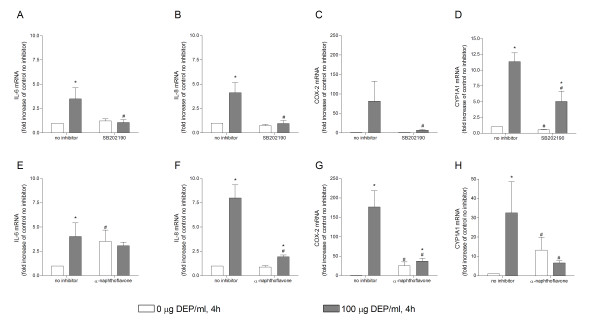
**Effect of inhibitors of p38 and CYP1A1 on DEP-induced mRNA expression of genes**. Effect of inhibitors of p38 (A-D) and CYP1A1 (E-H) on DEP-induced expression of IL-6 (A, E), IL-8 (B, F), COX-2 (C, G) and CYP1A1 (D, H) mRNA in cultures of human bronchial epithelial BEAS-2B cells. Cells were incubated with the p38 inhibitor (SB202190, 5 μM) or the CYP1A1 inhibitor (α-naphthoflavone, 25 μM) for 1 h, prior to addition of DEPs (0 and 100 μg/ml) for 4 h. Relative quantification of mRNA levels performed by QRT-PCR. Bars represent fold increase in mRNA relative to unexposed cells not treated with inhibitor detected in separate replicate experiments (means ± SEM, n = 4). * p < 0.05 exposed vs. unexposed cells. # p < 0.05 cells not treated with inhibitor vs. cells treated with inhibitor.

The involvement of p38 in DEP-induced mRNA expression of IL-6, IL-8, COX-2 and CYP1A1 was investigated by co-treatment of cells with the p38-inhibitor SB2020190. This treatment abolished the DEP-induced increase in the expression of IL-6, IL-8 and COX-2, but only partially reduced CYP1A1 (Figure 7A-D). The effects of the p38-inhibitor and of two other MAPK inhibitors, ERK (PD98059, 50 μM) and JNK (SP60015, 20 μM), on DEP-induced release of IL-6, was also investigated (data not shown). However, only the p38-inhibitor had an effect.

Co-treatment of cells with α-NF, a CYP1A1-inhibitor, proved to be very efficient in reducing the DEP-induced expression of IL-8 and COX-2 (Figure 7F, G). The inhibitory effect of α-NF on the DEP-induced expression of IL-6 was less evident (Figure 7E). As expected, α-NF reduced the DEP-induced expression of CYP1A1 (Figure 7H). However, α-NF also had stimulating effects on IL-6, COX-2 and CYP1A1 in cells not exposed to DEPs (Figure 7E, G, H). This stimulating effect may in part have camouflaged the effect of the inhibitor on the DEP-induced expression of IL-6.

**Figure 8:**
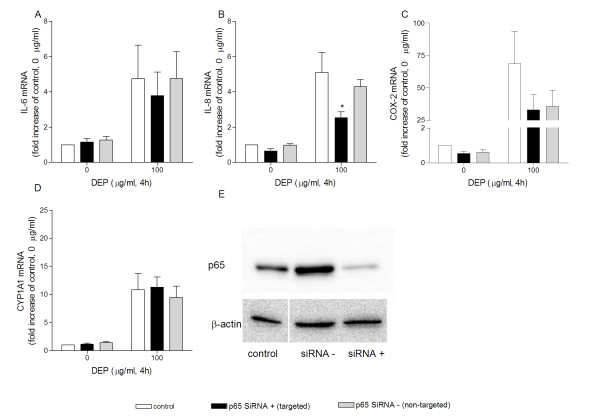
**Effects of siRNA against NF-κB (p65) on DEP-induced mRNA expression of genes**. Effects of siRNA against NF-κB (p65) on DEP-induced expression of IL-6 (A), IL-8 (B), COX-2 (C) and CYP1A1 (D) mRNA. Successful transfection was verified by Western analysis of p65-levels (E). Human bronchial epithelial BEAS-2B cells were exposed to DEPs (0 and 100 μg/ml) for 4 h. Relative quantification of cytokine mRNA levels was performed by QRT-PCR. Bars represent means ± SEM of fold increase relative to unexposed cells detected in separate experiments (n = 4). * p < 0.05 control vs. p65 siRNA + (targeted).

The involvement of NF-κB in the DEP-induced expression of the investigated genes was evaluated with siRNA for NF-κB p65. Apparently, p65 is not involved in the DEP-induced expression of CYP1A1, but might to a certain extent be involved in the expression of IL-8 and COX-2 (Figure 8A-D). Successful p65 gene silencing was confirmed with Western analysis (Figure 8E).

## Discussion

Studies with cell cultures, animals and human volunteers have shown that DEPs can induce production of various pro-inflammatory mediators in lung cells and tissue [16-18]. Due to associated compounds such as PAHs, DEPs are also well known for their carcinogenic properties [4], although a causal relationship between diesel exhaust exposure and lung cancer not yet has been conclusively demonstrated [19]. DEP-induced effects seem to involve CYP1A1-activity in the lung, which might be induced by PAHs in the organic fraction of the particles [6,20]. The possible relationship between the regulation of pro-inflammatory mediators and CYP1A1 has not been thoroughly investigated. In the present study we demonstrate that DEPs induced a pronounced expression of CYP1A1, at much lower concentrations than is needed to induce the inflammation-related genes IL-6, IL-8 and COX-2. Notably, inhibition of CYP1A1-activation clearly reduced the DEP-induced expression of IL-8 and COX-2, whereas its effect on IL-6 was less apparent. Furthermore, in line with findings from studies with human volunteers exposed to DEP [9], we detected DEP-induced activation of p38 as well as NF-kB/RelA. Whereas the DEP-induced increases in IL-8, COX-2 and IL-6 mRNA seemed dependent on p38, and IL-8 and COX-2 mRNA also on NF-κB, the increase in CYP1A1-expression seemed to be affected only moderately by p38 and not by NF-κB.

The marked induction of CYP1A1 at very low DEP-concentrations (0.025-25 μg/ml) is striking, occurring at approximately 1000-fold lower concentrations than the effect on IL-6, IL-8 and COX-2 expression, cytotoxicity and DNA-damage. This strongly suggests that the DEP-induced CYP1A1 response is exerted via mechanisms not involved in the other investigated end-points. Similarly, Vogel and co-workers have previously reported that DEP induces CYP1A1 mRNA expression at concentrations from 12.5 μg/ml in U937 macrophages, whereas IL-6 and COX-2 mRNA expression was increased at higher concentrations (from 50 μg/ml) [8]. However, in our study, the CYP1A1-increase occurred at much lower DEP-concentrations which were more different from the concentrations necessary to induce the inflammation-related genes. These differences may be attributed to differences in the applied DEP sample and/or cell type. In support of these findings, it has also been reported that soot particles (with adsorbed PAH), induce AhR-responsive genes (like CYP1A1) to a much larger magnitude than genes related to oxidative stress and inflammatory responses in murine lungs [21]. In our study, the increase in CYP1A1 expression occurred after two hours of DEP-exposure, before any significant cell death. At later time points, the highest DEP-concentrations used elicited significant cell death that was reflected in reduced CYP1A1 mRNA levels. However, at lower concentrations (25-100 μg/ml) the decline in CYP1A1 mRNA levels could not be due to cell death, but might be due to activation of other pathways, which antagonised the AhR-induced increase in CYP1A1 mRNA. It has for instance previously been demonstrated that activation of NF-κB may suppress the expression of CYP1A1 [22]. In support of this, we primarily detected activation of NF-κB after 4 h, upon exposure to 100 and 200 μg DEP/ml.

The concentration range used in this study is comparable to or lower than previous *in vitro *studies. However, compared to real world exposure levels, the concentrations used in such studies are generally considered to be on the high side. Exactly what levels epithelial lung cells are exposed to *in vivo*, is difficult to estimate due to the variations in and the complexity of inhaled particles' deposition patterns in the respiratory tract. Notably, inhaled particles are not symmetrically distributed throughout the respiratory tract, but tend to accumulate at so-called hot spots of deposition [23]. Li and colleagues have previously estimated that a biological relevant tissue culture concentration of DEP ranges from 0.2 to 20 μg/cm^2 ^[24]. In comparison, the applied concentration of 100 μg/ml in our study, corresponds to a concentration of 16 μg/cm^2^, which falls within this range. Notably, the DEP-induced CYP1A1-induction was apparent at much lower concentrations.

It is known that the AhR/ARNT pathway is important for modulation of inflammatory mediators, including IL-6, IL-8 and COX-2, especially after stimulation by agents like dioxins and PAH. This regulation is however complex, as activation of AhR has been suggested to differentially affect the induction of different cytokines, such as IL-6 and IL-8 via interaction with components of the NF-κB system [25]. Our findings suggest that AhR-activation also is important for DEP-induced increases in IL-8 and COX-2 levels, since their expression was almost abolished by α-NF, a classical AhR antagonist. For IL-6 the influence of DEP-induced AhR activation/CYP1A1 induction in the BEAS-2B cells was difficult to assess, as α-NF alone induced a marked response. It is known that α-NF, in addition to being an AhR-antagonist, also may act as a partial AhR-agonist [26]. Our findings indicate a different regulation of IL-6 versus IL-8, as the basal activity of the latter was not influenced by α-NF. Interestingly, with respect to this differential regulation, Vogel and co-workers demonstrated that the classical AhR-inducer, TCDD, induced IL-8 and COX-2, but not IL-6 in a human macrophage cell line [8]. Although AhR seems to contribute in the DEP-induced IL-8 and COX-2 mRNA response in the BEAS-2B cells, the increases in mRNA levels of the inflammation-related genes were detected at much higher concentrations than the activation of AhR. An interpretation of this may be that stimulation of other signalling pathways are most decisive for the inflammatory response, and that AhR may act as a permissive factor for IL-8 and COX-2 responses.

As expected, the stimulation of CYP1A1 mRNA levels seemed to depend on activation of the AhR, since α-NF inhibited the DEP-induced increase. In agreement with this, Vogel and co-workers reported that the CYP1A1-induction in macrophages by organic extracts of DEPs was partially reduced by AhR-inhibition, whereas the effect of the classical AhR-inducer, TCDD, was abolished [8]. P38 seems to alter AhR localisation and may therefore have an effect on CYP1A1 mRNA levels [27]. Our data indicate that p38-activation is involved in the induction of CYP1A1 mRNA, since p38-inhibition partially reduced CYP1A1 mRNA. In contrast to other MAPK-inhibitors, the p38 inhibitor (SB202190) is not an AhR agonist [28], and can therefore be used to investigate the role of p38 on CYP1A1 mRNA levels. At a high DEP concentration (200 μg/ml), that elicited strongly increased phosphorylation of p38, CYP1A1 mRNA levels were reduced to control levels. However, at lower DEP-concentrations (≤100 μg/ml), which induced higher CYP1A1 mRNA levels, the increase in p38-phosphorylation was low and likely negligible. This may suggest that the p38 effect on CYP1A1-expression may have been permissive only. In contrast, the DEP-induced expression of IL-6, IL-8 and COX-2 was abolished upon p38-inhibition, indicating a more direct role for p38 in the DEP-induced expression of these genes.

Though NF-κB seemed activated by DEP, as reflected by reduction in IκB and phosphorylation of p65 (RelA) in the classical NF-κB pathway, our data suggest that it did not influence CYP1A1 mRNA levels. This is not in agreement with other studies suggesting a negative involvement of RelA in complex with AhR in regulation of CYP1A1 levels and other P450 enzymes [22,25,29,30]. The interaction of components in the NF-κB-system with the AhR-pathway is very complex, and still not fully characterized. Interestingly, it has also been demonstrated that RelB, crucial in the alternative NF-κB pathway, may interact with the AhR, leading to a positive interaction with CYP1A1 [25]. Thus, the effect of DEP-induced NF-κB-activation on CYP1A1 induction may depend on the relative ability of DEP to trigger release of RelA versus RelB from their respective inhibitory counterparts (p50 and p52).

A crucial question is how AhR/NF-κB (RelA/RelB) interactions may influence the DEP-induction of inflammatory mediators. Upon TCDD exposure, RelA and RelB seem to interact very differently with AhR, inducing an inhibitory and stimulatory tonus, respectively, on cytokine induction [25]. Based on the outcome of the siRNA for NF-κB p65/Rel A in the present study, the classical NF-κB-pathway seems to play a certain role in the DEP-induction of IL-8, and possibly COX-2. However, as also indicated by the differential effect of α-NF on these genes, IL-6 again appeared as being regulated differentially from IL-8 and COX-2. Since activation of the classical NF-κB pathway usually seems to be important for of IL-8, IL-6, and COX-2 gene expression [31,32], we expected that siRNA against RelA would have had a somewhat greater and more similar effect on the DEP-induced expression of these genes. It may however be speculated that the siRNA also reduced the formation of inhibitory AhR/RelA complexes, and thereby caused a less pronounced inhibition of the expression of the investigated genes. Another possibility is that the relative role of NF-κB - versus AP-1-mediated responses depends on the composition of the DEP-sample used. Recently, Tal and colleagues reported that DEPs with high and low organic content induced IL-8 expression via different regulatory pathways in BEAS-2B cells [33]. The low organic component DEP required NF-κB-activation whereas the high organic DEP mediated its effect via a NF-κB-independent, but AP-1-dependent mechanism [33]. Formation of ROS may be involved in modulation of activity of both these transcription factors [34-36]. Furthermore, DEP and associated PAHs have been reported to trigger ROS, which is thought to be crucial in DEP-induced cytokine formation, cytotoxicity and DNA damage [37,38]. However, to what extent ROS-effects are involved in the NF-κB -/p38-independent CYP1A1-mediated pathway, or the NF-κB/p38-dependent pathway mediating the IL-8 and COX-2 expression, needs to be further addressed.

Our data, with effects of α-NF on DEP-induced induction of CYP1A1, IL-8 and COX-2 suggest that the organic fraction of the particles may be of importance. To what extent the measured PAHs (Additional file 1) are responsible for the DEP-induced effect on CYP1A1 expression, needs to be further studied. It was recently reported that DEPs of varying organic content induced IL-8 expression with varying efficacy, with the high organic content DEPs being the most potent [33]. However, DEPs with low organic content also induced IL-8 expression, indicating that the organic content is not the sole determinant of the biological potency of a particle [33]. The metal content has for instance also been identified as influential components for particle-induced effects [39,40]. Furthermore, even if PAHs seem important for AhR/CYP1A1-linked processes, this group of components does not need to be the major determinant for the induction of IL-6, IL-8 and COX-2. Interestingly, heavy metals are also reported to induce expression of CYP1A1, and activation of NF-κB and AP-1 signalling pathways are suggested to be directly involved [30]. Although intriguing, identification of the causative component of the DEP-induced responses was not the aim of the present study. These findings may, however, be followed-up by including several samples of DEPs with contrasting contents of PAHs and metals.

## Conclusions

**Figure 9:**
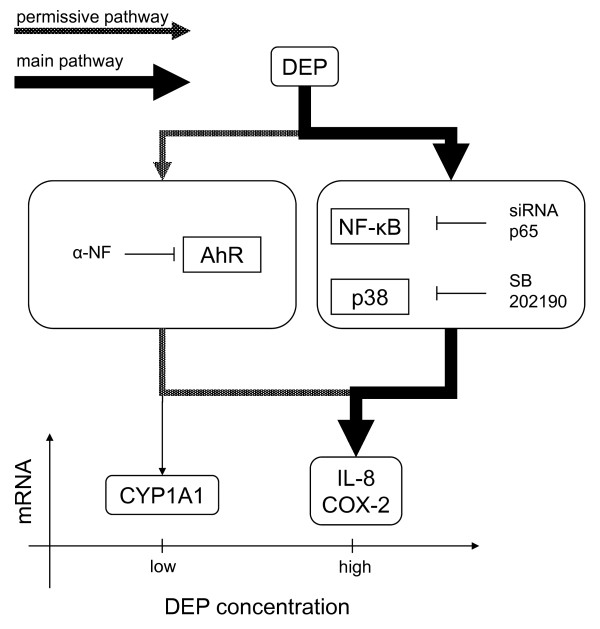
**Proposed model for DEP-induced expression of IL-8, COX-2 and CYP1A1, based on the presented findings**. DEP-induced mRNA expression of CYP1A1 in BEAS-2B cells occurs at much lower DEP-concentrations than the concentrations necessary to induce mRNA expression of IL-8 and COX-2. Activation of AhR and expression of CYP1A1 mRNA seem essential in facilitating the DEP-induced expression of IL-8 and COX-2 mRNA via a permissive pathway (required, but not decisive for the strength of the response), not involving p38 and NF-κB/p-65. However, the p38 and p65 pathways seem crucial in DEP-induced expression of COX-2 and IL-8 mRNA, via a major pathway.

The present study indicates that DEPs induce CYP1A1, IL-6, IL-8 and COX-2 in BEAS-2B-cells. The DEP-induced CYP1A1-expression occurred at much lower DEP-concentrations than the concentrations necessary to induce expression of IL-6, IL-8 and COX-2, and cytotoxicity and DNA-damage. The activation of AhR/CYP1A1 expression seems essential in facilitating the DEP-induction of the pro-inflammatory mediators via a permissive mechanism not involving p38 and NF-κB/p65 (Proposed model, Figure 9). The p38 and p65 pathways, however, seem crucial in DEP-induction of COX-2 and IL-8, via another major pathway (Figure 9). Notably, DEP-induced IL-8 and COX-2 seem to involve different pathways than IL-6, possibly triggered by different components in DEP. Our study indicates that expression of CYP1A1 may represent a sensitive biomarker for DEP-induced effects. Further studies should emphasize this, by examining whether CYP1A1 has a rate-limiting role in the toxic mechanism of different types of DEPs and combustion particles with contrasting contents of components.

## Methods

### Chemicals/reagents

LHC-9 cell culture medium was purchased from Invitrogen (Carlsbad, CA, USA) and PureCol™ collagen from Inamed Biomaterials (Fremont, CA, USA). All real-time PCR reagents and TaqMan probes/primers were purchased from Applied Biosystems (Foster City, CA, USA). Inhibitors, including SB202190 (4-[4-fluorophenyl]-2-[4-hydroxyphenyl]- 5-[4-pyridyl]1H-imidazole), PD98059 (2-amino-3-methoxyflavone) and SP600125 (anthrax[1,9-cd]pyrasol-6(2H)-one), were purchased from Calbiochem-Novabiochem Corporation (La Jolla, CA, USA), and the CYP1A1 inhibitor, α-naphthoflavone (α-NF) from Sigma-Aldrich (St. Louis, MO, USA). Specific antibodies against phospho- and total p38, JNK1/2 and p65, were obtained from Cell Signalling Technology Inc. (Beverly, MA, USA) and antibodies against phospho- and total ERK1/2 from Santa Cruz Biotechnology Inc. (Santa Cruz, CA, USA). β-actin was purchased from Sigma-Aldrich (St. Louis, MO, USA), and mild antibody stripping solution^® ^from Chemicon International (Termecula, CA, USA). NF-κB p65 siRNA and control siRNA (Human) SignalSilence^® ^kits were purchased from Cell Signaling Technology (Beverly, MA, USA) and HiPerFect^® ^Transfection Reagent from QIAGEN (Hilden, Germany). All other chemicals were purchased from commercial sources at the highest purity available.

### Collection and characterisation of diesel exhaust particles

DEPs were generated from an unloaded diesel engine (Deutz, 4 cylinder, 2.2 L, 500 rpm) using gas oil (Petroplus Refining Teesside Ltd., United Kingdom). More than 90% of the exhaust was shunted away through the main exhaust, and the remaining part was diluted with air, passed through an impactor with a cutoff of 0.1 μm, and fed at 75 L/min into a 2 m^3 ^chamber at steady-state concentration of approximately 300 μg/m^3^. The DEPs used in the present study were collected from the main diesel exhaust after the unloaded diesel engine had run for 8 weeks, and the chamber study was terminated. Particles and volatiles deposited onto the walls of the main exhaust were carefully taken from the inner pipes wall into a clean beaker. This soot was carefully mixed and divided over several pre weighed aliquots. The aliquots were weighed again to determine the particulate matter mass. The vials were labelled and stored at -80°C until chemical analyses (Additional file 1) and in vitro experiments.

The cytotoxic potential of the diesel sample described above was compared with the cytotoxic potential of the commercially available Standard Reference Material 1650a (SRM 1650a), obtained from the Office of Standard Reference Materials, National Institute of Standards and Technology (NIST), Gaithersburg, MD, USA. SRM 1650a has been collected from heavy duty diesel engines representative for the early 1980's and is intended for use as model for heavy duty diesel engine particulate emissions. According to the Certificate of Analysis [41], the content of for instance the classical PAH B[a]P is lower in SRM1650a (1.17 mg/kg), compared to the collected DEP sample (4.739 mg/kg, Additional file 1).

Particles were suspended in cell exposure medium (2 mg/ml in LHC-9 medium) and stirred overnight in room temperature before exposure of cells.

### Culture of cells

BEAS-2B cells, a SV40-transformed human bronchial epithelial cell line was purchased from the European Collection of Cell Cultures (ECACC, Salisbury, UK). Cells were maintained in LHC-9 medium in collagen (PureCol™)-coated flasks in a humidified atmosphere at 37°C with 5% CO_2_, with refreshment of medium every other day. One day prior to exposure, BEAS-2B cells (passages 17-68, and with new batch passages 4-60) were plated into collagen (PureCol™)-coated 35 mm 6-well culture dishes (460.000 cells/well). In case of Western analysis, cells were plated into collagen (PureCol™)-coated 90 mm culture dishes (1.500.000 cells/dish), two days prior to exposure.

### Exposure of cells

Depending on the experiment, cells were incubated with various concentrations of DEP (0-400 μg/ml) for 2 or 4 h (immunoblotting), for 2, 4, 8 and 24 h (mRNA expression) or for 2, 4, 6 and 24 h (protein release and cell death). In all experiments control cells were exposed to medium that had been subjected to the same stirring procedure as the particle suspension.

Where applicable, cell cultures were treated with the CYP1A1 inhibitor, α-naphthoflavone (α-NF) (25 μM), or with the MAPK-inhibitors PD98059 (25 μM), SB202190 (5 μM) and SP600125 (20 μM) for 1 h, before particles were added. The applied concentrations of the inhibitors were based on experience from previous experiments conducted with these inhibitors on BEAS-2B cells in our laboratory.

Total exposure volume was 1.5 ml and 10 ml in 6-well 35 mm and 90 mm cell culture dishes, respectively. From this, it may be calculated that an applied particle concentration of 100 μg/ml corresponds to a concentration of 16 μg/cm^2^, if all the suspended particles deposit on the cells present on the surface of the culture dishes.

### Examination of DEP-induced cell damage

Following exposure, floating and attached cells were stained with propidium iodide (PI; 10 μg/ml) and Hoechst 33342 (5 μg/ml) for 30 min to determine plasma membrane damage. Cell morphology was evaluated using a Nikon Eclipse E 400 fluorescence microscope. Cells with clearly condensed and/or fragmented nuclei were counted as apoptotic, PI-stained cells as necrotic, and non-apoptotic cells excluding PI as viable cells. The percentage of apoptotic and necrotic cells were determined as a fraction of the total number of counted cells.

### Examination of DEP-induced DNA damage

The experimental protocol and the results are enclosed in online Additional file 2.

### Examination of DEP-induced mRNA expression

After exposure, cellular RNA was isolated from cells according to the supplier's recommendations, using the Absolutely RNA™ RT-PCR Miniprep kit (Stratagene, La Jolla, CA). Subsequently, mRNA in each sample was reverse-transcribed into cDNA on a PCR system 2400 (Perkin Elmer) by using a High Capacity cDNA Archive Kit from Applied Biosystems.

The screening of multiple genes (CCL5/-17/-20/-26, CXCL5/-10/-16, ALOX5, CSF2, IL-1β, IL-6, IL-8, IL-10, IL-13, IL-18, IL-33, IL-1RN, PTGS2 (COX-2), TGF-β1 and TNF-α) was carried out by application of a customized real-time PCR array (RT^2 ^Profiler™ PCR Array Customized Product, MD USA) and the Applied Biosystems 7500 Real-Time PCR System. The selection of genes to be included in the array was based on a previous screen of 82 inflammation-related genes after exposure to components commonly associated with combustion particles [15]. The expression of each gene within each sample was normalised against β-actin and expressed relative to the control sample using the formula 2-(ΔΔCt), in which ΔΔCt = (Ct mRNA - Ct β-actin rRNA)_sample _- (Ct mRNA - Ct, β-actin rRNA)_control sample_. The array also contained a positive PCR control and a genomic DNA control, which both were within the advised Ct-ranges.

Further analysis of IL-6, IL-8, COX-2 and CYP1A1 mRNA levels was also performed by using the Applied Biosystems 7500 Real-Time PCR System, but in this case with pre-designed TaqMan Gene Expression Assays (18S, Hs99999901_s1; IL-6, Hs00174131_m1; IL-8, Hs00174103_m1; COX-2, Hs01573471; CYP1A1, Hs00153120_m1) and TaqMan Universal PCR Master Mix. The expression of each gene within each sample was normalised against 18S rRNA and expressed relative to the control sample using the formula 2-(ΔΔCt), in which ΔΔCt = (Ct mRNA - Ct 18S rRNA)sample - (Ct mRNA - Ct, 18S rRNA)control sample.

### Quantification of DEP-induced cytokine release

After exposure, cell culture supernatants were collected and centrifuged twice for removal of dead cells (300 × g) and particles (8000 × g), and stored at -70°C until cytokine analysis. Concentrations of IL-6 and IL-8 in cell culture supernatants were determined by ELISA (R&D Systems, Minneapolis MN, USA), according to the manufacturer's manual. The increase in colour intensity was measured and quantified using a plate reader (TECAN Sunrise, Phoenix Research Products, Hayward, CA, USA) with software (Magellan V 1.10). Cytokine concentrations are expressed in pg/ml.

### Examination of MAPK- and p65 phosphorylation and degradation of IκBα

DEP-induced phosphorylation of MAPKs and p65, and degradation of IκBα, were analysed by Western analysis. After exposure, cell culture medium was removed and the dishes were immediately rinsed with ice-cold PBS, and stored at -70°C until further processing. Frozen cells were thawed, harvested and sonicated in lysis buffer (20 mM Tris-HCL, pH = 7.5, 150 mM NaCl, 1 mM EDTA, 1 mM EGTA, 2.4 mM Na-pyrophosphate, 1.0 mM orthovanadate, 1 mM NaF, 21 μM leupeptin, 1.5 μM aprotinin, 15 μM pepstatin A and 1% Triton-X) prior to protein determination using the BioRad DC Protein Assay (BioRad Life Science, CA, USA). Subsequently glycerol, β-mercaptoethanol and SDS were added to all samples, whereas final sample protein concentrations were adjusted by adding more lysis buffer. Proteins (12.5-25 μg/well) from whole-cell lysates were separated by 10% SDS-PAGE and blotted onto nitrocellulose membranes. To ensure that the protein levels of each well were equal, Ponceau-staining was used for loading control. The membranes were then probed with antibodies for the respective phosphorylated kinases (p-ERK1/2, p-JNK1/2, p-p38, p-p65) or with IκBα prior to incubation with horseradish peroxidase-conjugated secondary antibodies. The blots were developed using the Super-Signal^® ^West Dura chemoluminiscence system (Pierce, Perbio Science, Sweden) according to the manufacturer's instructions. Finally, the membranes were stripped by incubation for 15 min at room temperature with mild antibody stripping solution, and re-probed with β-actin, or with the total amount of the respective kinases and p65. Optical quantification of the protein bands were performed by using the KODAK 1D Image Analysis Software.

### Suppression of p65 by siRNA

The involvement of NF-κB in the DEP-induced expression of the investigated genes was evaluated with siRNA for NF-κB p65. For those experiments cells were plated into 35 mm collagen-coated 6-well culture dishes at a density of 200.000 cells/well, and immediately treated either with p65-siRNA (10 nM) and HiPerfect reagent (6 μl/well), or with non-targeting control siRNA (10 nM) and HiPerfect reagent (6 μl/well). Medium was changed (refreshed) after 24 h, and on the day of exposure (48 h after transfection). The silencing of NF-κB p65 expression was confirmed by Western analysis 48 h after transfection.

### Calculations and statistical analysis

The results presented in Figures 1 and 4 were analysed statistically by application of a one-way analysis of variance (ANOVA) with Dunnett's multiple comparison test. Results presented in Figures 3, 5, 7 and 8 were analysed statistically by application of a two-way ANOVA with Bonferroni post tests. Statistical analysis of the data presented in Figure 3, 4, 5, 7 and 8 was carried out on log-transformed data. Statistical analyses were performed using GraphPad Prism software (version 4.03, Inc., San Diego, CA). p < 0.05 was considered to reflect statistically significant differences.

## Competing interests

The authors declare that they have no competing interests.

## Authors' contributions

AIT planned and conducted the toxicological analyses, was involved in the interpretation of the results, and drafted the manuscript. FRC was responsible for collection and characterisation of the diesel sample and was involved in the interpretation of results and final manuscript preparation. PS was involved in the planning of the study, interpretation of the results, and the final manuscript preparation. MR was involved in interpretation of results and manuscript preparation. ML was involved in the planning of the study, interpretation of the results and manuscript preparation. All authors have read and approved the final manuscript.

## Supplementary Material

Additional file 1 **Chemical analysis of the diesel engine exhaust particles (DEPs)**. Analytical procedure for and outcome of the analysis of the content of elemental and organic carbon, anions (nitrate and sulphate), PAHs and metals of the DEPs collected to be used in the present study. Click here for file

Additional file 2 **DEP-induced DNA-damage**. Description of the methods for and the results of the examination of DEP-induced DNA-damage. Click here for file
