# Supplementary material for: Diesel exhaust particles induce CYP1A1 and pro-inflammatory responses via differential pathways in human bronchial epithelial cells
Source: Part Fibre Toxicol. 2010 Dec 16;7:41. doi: 10.1186/1743-8977-7-41 (PMC3012014; doi:10.1186/1743-8977-7-41)
Supplement: Additional file 1 — Chemical analysis of the diesel engine exhaust particles (DEPs). Analytical procedure for and outcome of the analysis of the content of elemental and organic carbon, anions (nitrate and sulphate), PAHs and metals of the DEPs collected to be used in the present study. [file 1743-8977-7-41-S1.DOC]

Additional file 1. **Chemical analysis of the diesel engine exhaust particles (DEPs)**

The chemical analysis of the DEP sample was conducted by the Netherlands Organisation for Applied Scientific Research (TNO) in the Netherlands. The applied methods of analysis are methods validated by TNO and are based on existing International guidelines. All the analysis results reported were corrected for laboratory procedure blanks.

**Elemental carbon/Organic carbon (EC/OC) and anions (nitrate and sulphate)**

To determine the content of organic carbon (212 µg/mg dust), elemental carbon (38 µg/mg), total carbon (250 µg/mg), NO_3_ (0.93 µg/mg) and SO_4_ (7.4 ug/mg), 36.5 mg of the diesel sample was used.

Samples were brought into suspension in Milli-Q-water by sonication. A part of the waterlayer was diluted and analysed for anions. For EC/OC a known volume of suspended material was added to a quartzfiber filter and dried at room temperature. A punch of the dried quartzfiber filter was used for EC/OC analysis. The analysis of EC/OC is based on the thermal optical method as described in the American Standard Method NIOSH 5040. The EUSAAR 2 protocol was used for the temperature settings. OC was removed from the filter in the temperature range of 200-650 °C in a non-oxidising carrier gas (Helium). Subsequently EC was removed in the temperature range of 500-850 °C, making use of a mixture of helium and oxygen. The CO_2_ originating is then converted to methane and detected by flame ionisation detection (FID). Correction for pyrolysis was carried out by measurement of light transmission. Quantification took place based on a gas mixture of 5% methane in helium. Sucrose was used as a control standard. The total uncertainty of the method amounts 31%.

Nitrate and sulphate were analysed by Ion chromatography with detection based on suppressed conductivity. The measurement uncertainty of the analytical method amounts 6 and 8% for respectively nitrate and sulphate.

**PAHs**

The DEP sample was extracted by means of sonication with hexane and subsequently fractioned into two fractions using a silica column, in order to separate the hopanes from the PAHs. Prior to extraction, two internal standards were applied to the sample (a mixture of the 16 deuterated EPA-PAH components and d50 -n-tetracosane). The extracts were concentrated by evaporation under nitrogen to a volume of 1 ml. For the correction of the injection volume, tetrachloronaphthalene (TCN) and PCB209 was added to the final extract. The 16 EPA (US Environmental Protection Agency) PAHs were analysed by means of gas chromatography in combination with mass spectrometric detection in electron impact mode (GC/MS EI). The analyses were conducted on an Agilent 6890/5973N GC/MS. Component identification was based on retention time and qualifier ion ratios. Quantification was conducted based on the highest characteristic qualifier ion using the internal standard technique. The total uncertainty of the method varied per component from 20 to 26% for respectively (semi)-volatile and particle bound PAHs. The following PAH components were analysed: naphthalene, acenaphthalene, acenaphtene, fluorene, phenanthrene, anthracene, fluoranthene, pyrene, benzo[a]anthracene, chrysene, benzo[b]fluoranthene, benzo[k]fluoranthene benzo[a]pyrene, indeno[1,2,3-CD]pyrene, dibenzo[a,h]anthracene and benzo[g,h,i]perylene. Table 1 shows the content of these PAHs in the DEP sample.

Table 1. PAH content of the DEPs

| **PAH** | **ng/g** | **PAH** | **ng/g** | **PAH** | **ng/g** |
| --- | --- | --- | --- | --- | --- |
| acenaphthene | 4347 | benzo[ghi]perylene | 297 | indeno[123-cd]pyrene | 574 |
| acenaphthylene | 314 | benzo[k]fluoranthene | 2304 | naphthalene | 7668 |
| anthracene | 4081 | chrysene | 44753 | phenanthrene | 113202 |
| benzo[a]anthracene | 5565 | dibenzo[ah]anthracene | 147.5 | pyrene | 82912 |
| benzo[a]pyrene | 4739 | fluoranthene | 29604 |  |  |
| benzo[b]fluoranthene | 4577 | fluorene | 14195 |  |  |

**Metals**

Samples were digested during 1 hour with diluted nitric acid and diluted hydrogen peroxide using a heating block. The heavy metals were analysed by high resolution inductively coupled plasma mass spectrometry (ICP-MS). The analysis was conducted on a Thermo element 2 ICP/MS. Component identification was based on the specific mass of the corresponding element. Quantification took place with the use of a calibration standard, which was validated with an external standard. Table 2 shows the content of different metals in the DEP sample.

Table 2. Metal content of the DEPs

| **Metal** | **ng/mg** | **Metal** | **ng/mg** | **Metal** | **ng/mg** | **Metal** | **ng/mg** |
| --- | --- | --- | --- | --- | --- | --- | --- |
| Aluminium | 415 | Europium | 0.02 | Molybdenum | 0.5 | Strontium | 7.7 |
| Antimony | < | Gadolinium | 0.07 | Neodymium | 0.31 | Sulfur | 7035 |
| Arsenic | 0.22 | Germanium | < | Nickel | 9.0 | Tantalum | < |
| Barium | 20 | Gold | < | Niobium | < | Terbium | < |
| Beryllium | 0.01 | Hafnium | 0.07 | Palladium | < | Thallium | < |
| Bismuth | 0.02 | Holmium | 0.27 | Platinum | < | Thorium | < |
| Boron | 2.0 | Indium | 0.03 | Potassium | 90 | Thulium | 0 |
| Cadmium | 0.06 | Iridium | < | Praseodymium | 0.08 | Tin | 3 |
| Calcium | 1548 | Iron | 666 | Rhenium | < | Titanum | 88 |
| Cerium | 0.7 | Lanthanum | 0.33 | Rubidium | 0.22 | Uranium | 0 |
| Cesium | < | Lead | 6.6 | Ruthenium | < | Vanadium | 2 |
| Chromium | 6 | Lithium | < | Samarium | 0.07 | Ytterbium | 0.13 |
| Cobalt | 0.16 | Lutium | 0.06 | Scandium | < | Yttrium | 0.26 |
| Copper | 9 | Magnesium | 419 | Selenium | 1 | Zinc | 4644 |
| Dysprosium | 0.12 | Manganese | 32 | Silicon | 14 | Zirkonium | 1.4 |
| Erbium | 0.32 | Mercury | < | Sodium | 251 |  |  |

< not detectable
