# Supplementary material for: Diesel exhaust particles induce CYP1A1 and pro-inflammatory responses via differential pathways in human bronchial epithelial cells
Source: Part Fibre Toxicol. 2010 Dec 16;7:41. doi: 10.1186/1743-8977-7-41 (PMC3012014; doi:10.1186/1743-8977-7-41)
Supplement: Additional file 2 — DEP-induced DNA-damage. Description of the methods for and the results of the examination of DEP-induced DNA-damage. [file 1743-8977-7-41-S2.DOC]

Additional file 2. **DEP-induced DNA-damage**

# Method

DEP-induced DNA damage was investigated by the comet assay, carried out as previously described [1]. In brief, media were removed after exposure and cells were trypsinized and resuspended in medium. Trypsinized cells were diluted in low melting point agarose (to a final concentration of 0.75%) and moulded onto GelBond films attached to plastic frames to facilitate subsequent treatment steps. Shortly after moulding, films were immersed in lysing solution. After lysis overnight, films were rinsed quickly in distilled water and immersed in enzyme buffer (10 + 50 min at 4 °C). Subsequently films were incubated in pre-warmed enzyme buffer with 0.2 mg/ml bovine serum albumin for 60 min at 37 °C, with or without a bacterial Fpg enzymic extract. Enzyme treatment was followed by unwinding in electrophoresis buffer (5 + 35 min at 4 °C), and electrophoresis (20 min at 8 °C) at 0.8 V/cm (total voltage 20 V, 300 mA). After neutralization (3x5 min), films were rinsed in water, fixed in ethanol (90 min) and dried until further analysis. Dried films were rehydrated and stained in Tris-EDTA buffer (20 min) with SYBERGold® (1:12500 dilution) for 20 min, before visualization and scoring of comets using the image analysis software ‘Comet assay IV’ (Perceptive Instruments Ltd, Haverhill, Suffolk, UK) and an Olympus BX51 fluorescence microscope (Japan), with an A312f camera (Basler Vision Technologies, Ahrensburg, Germany).

# Results:

To limit the influence of cell death on the DNA damage results, exposure time points of 2 and 4 h were chosen. DNA damage was only apparent at high DEP-concentrations, 200 μg/ml and 400 μg/ml, with and without Fpg enzyme treatment, respectively (Fig. 1). These concentrations are much higher than the concentrations at which maximal expression of both CYP1A1 and the inflammation-related genes were detected (see main manuscript).


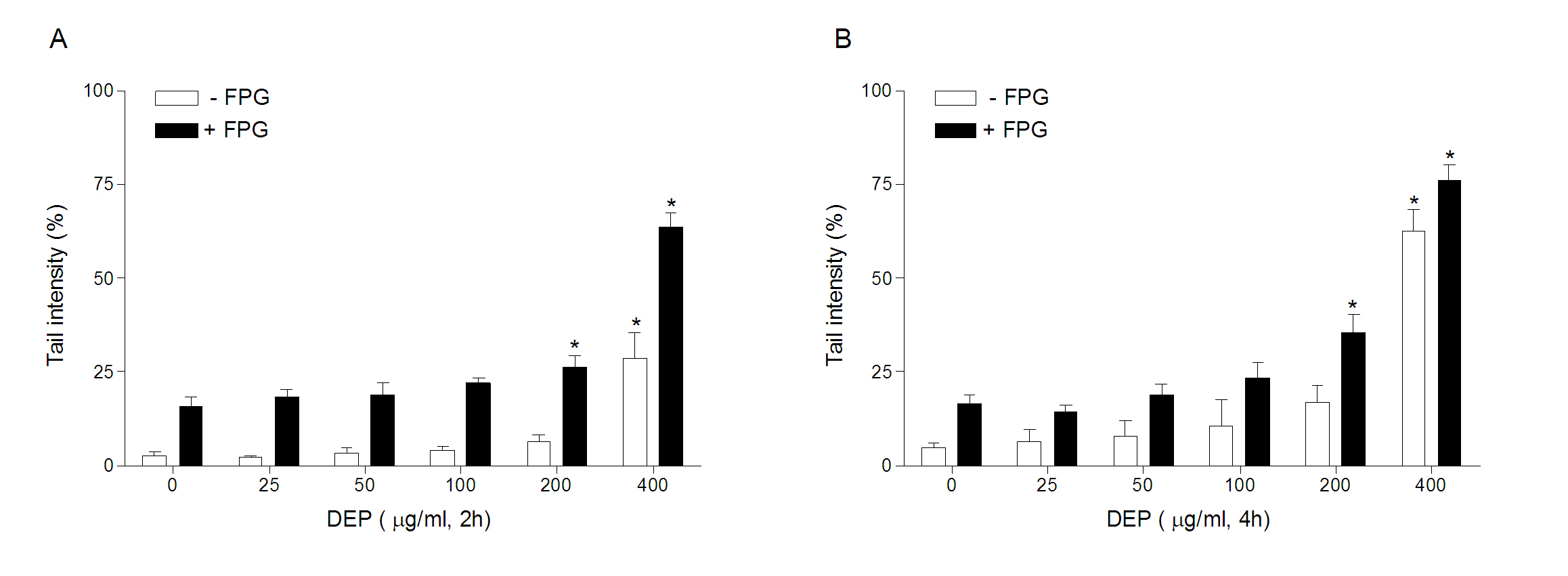


## Figure 1 - Concentration-dependent DEP-induced DNA damage after 2 h exposure (A) and 4 h exposure (B).

Human bronchial epithelial BEAS-2B cells were exposed to increasing concentrations of DEPs (0-400 µg/ml) and analysed with or without treatment with Fpg enzyme by single-cell gel electrophoresis. Bars represent means ± SEM of counted cells detected in separate experiments (n=3). * p < 0.05; exposed vs. unexposed cells.

References

1. Hansen SH, Olsen AK, Søderlund EJ, Brunborg G: **In vitro investigations of glycidamide-induced DNA lesions in mouse male germ cells and in mouse and human lymphocytes.** *Mutation Research/Genetic Toxicology and Environmental Mutagenesis* 2010, **696:**55-61.
